# Bacteria‐Based Living Probes: Preparation and the Applications in Bioimaging and Diagnosis

**DOI:** 10.1002/advs.202306480

**Published:** 2023-11-30

**Authors:** Hejin Jiang, Zhenping Cao, Ying Liu, Rui Liu, Yan Zhou, Jinyao Liu

**Affiliations:** ^1^ Shanghai Key Laboratory for Nucleic Acid Chemistry and Nanomedicine Institute of Molecular Medicine State Key Laboratory of Systems Medicine for Cancer Renji Hospital School of Medicine Shanghai Jiao Tong University Shanghai 200127 China; ^2^ Department of Radiology Renji Hospital School of Medicine Shanghai Jiao Tong University Shanghai 200127 China

**Keywords:** bacteria, bioimaging, diagnosis, probes, theranostic

## Abstract

Bacteria can colonize a variety of in vivo biointerfaces, particularly the skin, nasal, and oral mucosa, the gastrointestinal tract, and the reproductive tract, but also target specific lesion sites, such as tumor and wound. By virtue of their prominent characteristics in motility, editability, and targeting ability, bacteria carrying imageable agents are widely developed as living probes for bioimaging and diagnosis of different diseases. This review first introduces the strategies used for preparing bacteria‐based living probes, including biological engineering, chemical modification, intracellular loading, and optical manipulation. It then summarizes the recent progress of these living probes for fluorescence imaging, near‐infrared imaging, ultrasonic imaging, photoacoustic imaging, magnetic resonance imaging, and positron emission tomography imaging. The biomedical applications of bacteria‐based living probes are also reviewed particularly in the bioimaging and diagnosis of bacterial infections, cancers, and intestine‐associated diseases. In addition, the advantages and challenges of bacteria‐based living probes are discussed and future perspectives are also proposed. This review provides an updated overview of bacteria‐based living probes, highlighting their great potential as a unique yet versatile platform for developing next‐generation imageable agents for intelligent bioimaging, diagnosis, and even therapy.

## Graphical abstract

Here, it introduces the strategies for preparing bacteria‐based living probes, summarize their recent progress in different imaging modalities, and highlight its applications in bioimaging and diagnosis of bacterial infections, cancer, and intestine‐associated diseases. This review provides an updated overview of bacteria‐based living probes and elaborates its current challenges and future perspectives, which can inspire the development of next‐generation imageable probes.

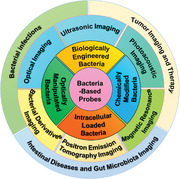

## 1 Introduction

As an important technique to visualize and elucidate the tissue structure and various physiological functions of organisms, bioimaging has been widely used to preciously diagnose and monitor the physiological and pathological processes of organisms in real time.^[^ ^1^ ^]^ Bioimaging techniques, including optical imaging, ultrasonic imaging, photoacoustic imaging, magnetic resonance imaging (MRI), and positron emission tomography (PET) imaging, have been extensively applied in the initial diagnostic imaging of various diseases. Given their unique advancements, bioimaging techniques also boost the development and popularization of corresponding bioimageable probes, such as optical probes, ultrasonic probes, photoacoustic probes, and radioactive probes. Normally, the characterizations of probes in sensitivity, specificity, and response speed can affect the imaging performance directly. In particular, probes can be utilized in a non‐invasive way, and also be combined with therapeutic agents to simultaneously achieve real‐time imaging and disease diagnosis and treatment.^[^ ^2^, ^3^ ^]^

As well known, by virtue of their unique properties in biocompatibility, motility, editability, and specific site targeting ability, bacteria have been developed as versatile living probes for bioimaging, diagnosis, and therapy of different diseases, such as bacterial infections, cancers, and intestinal diseases.^[^ ^4^, ^5^, ^6^ ^]^ Given their editable characteristic, bacteria can be equipped with various signal molecules both inside and outside via either genetic modification or surface decoration.^[^ ^7^ ^]^ To obtain specificity, the engineered bacteria can be prepared by combining bacteria with signal molecules in different ways, including biological methods (such as genetic engineering and metabolic labeling), chemical modification (such as non‐covalent bonding and covalent chemical conjugation), bacterial intracellular loading, and optical manipulation.^[^ ^8^, ^9^, ^10^, ^11^, ^12^ ^]^ Furthermore, bacteria can sense and respond to the physiological environments (for example, the intestine) and migrate to the region of diseases (such as tumor and other infection sites).^[^ ^13^, ^14^ ^]^ Therefore, these engineered bacteria‐based living probes can accumulate and even colonize specific regions to act as probes for bioimaging and diagnosis of diseases.

**Figure 1:**
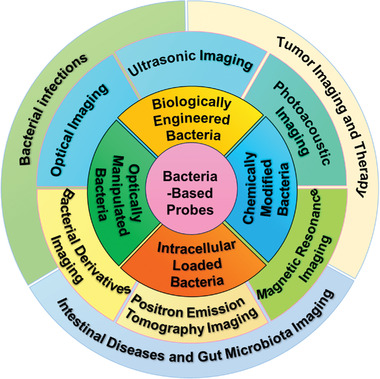
Construction strategies and applications of bacteria‐based living probes for bioimaging and diagnosis of diseases.

In this review, we aim to provide a snapshot of bacteria‐based living probes and the applications in biomedicine. We systematically summarize their recent advances as living imaging and diagnostic probes for various diseases. The preparation strategies of bacteria‐based living probes are first introduced and then different types of these living probes for bioimaging and diagnosis are discussed. Lastly, we highlight the promising potential and success of these bacteria‐based living probes in the diagnosis and treatment of different diseases, specifically focusing on bacterial infections, cancers, and intestine‐associated diseases (**Figure** **1**).

## 2 Preparation of Bacteria‐Based Living Probes

**Figure 2:**
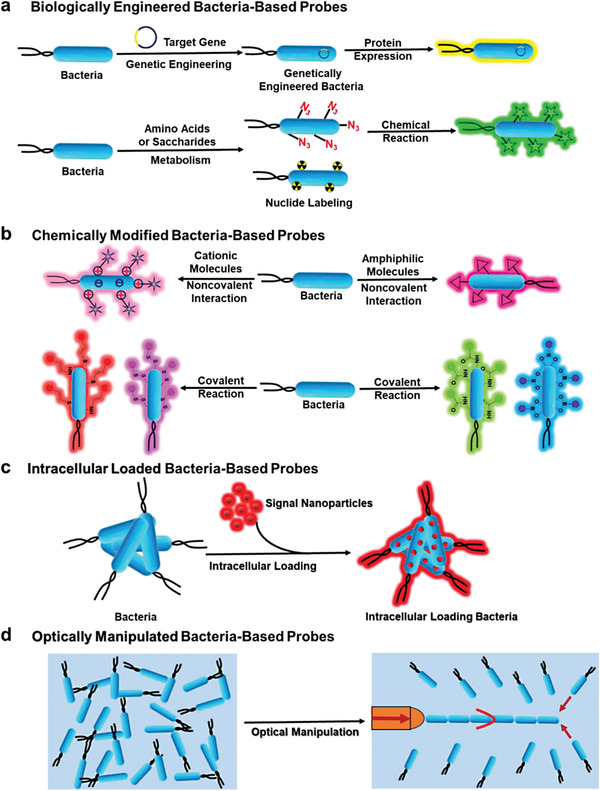
Construction strategies of bacteria‐based living probes. a) Biological engineering; b) Chemical modification; c) Intracellular loading; d) Optical manipulation.

Bacteria are ubiquitous and abundant in our surroundings, and the changes in biological microenvironments can cause influence on bacterial behaviors. For example, under unfavorable culture conditions, the substances in bacterial cell walls and cell membranes, such as proteins and carbohydrates, can be affected. Bacteria can also responsively increase the production of bacterial outer membrane vesicles (OMVs) and the formation of biofilms to fight against such environments, and even change the secretion of metabolites for better survival. ^[^ ^15^ ^]^ More attractively, bacteria exist widely on/in healthy tissues (such as the skin, oral cavity, the gastrointestinal (GI) tract) and unhealthy sites (including infection sites and tumors).^[^ ^14^, ^16^ ^]^ These bacteria can regulate the distribution of flora by communicating with the surrounding bacteria, and also affect the physiological functions of human cells and tissues through metabolites or direct interaction.^[^ ^17^ ^]^ Moreover, relying on these direct and indirect interactions between bacteria and the host, bacteria can be an appealing candidate as functional bacteria‐based living probes for bioimaging and diagnosis of various diseases associated with the host. Thus, how to introduce bioimaging signal molecules to prepare bacteria‐based living probes becomes the key to efficient real‐time diagnostic imaging. With the advancement of interdisciplinarity, the construction strategies of bacteria‐based living probes have expanded to chemistry, materials, biology, and other disciplines in the past years. Three main methods including biological engineering, chemical modification, intracellular loading, and optical manipulation have been adopted in designing bacteria‐based living probes with tunable functions (**Figure** **2**).

### 2.1 Construction of Bacteria‐Based Living Probes by Biological Engineering

The vast majority of bacteria are naturally absence of bioimaging signal molecules, and cannot be used directly for bioimaging and diagnosis of diseases. With the development of biological engineering technology, such as genetic engineering, synthetic biology, and metabolic engineering, diverse bacterial strains exhibit promising potential to act as living probes in vivo.^[^ ^18^, ^19^ ^]^ By virtue of biological engineering technology, different types of signal molecules can be integrated into bacteria smoothly without influencing their natural function and integrity. To date, genetic engineering and metabolic labeling represent the two most widely used technologies for integrating bacteria with signal molecules via biological engineering. For example, by integrating exogenous genes into bacterial genome or transferring plasmids that are capable of expressing fluorescent proteins, bacteria can be genetically engineered to be living probes for fluorescence imaging. Because of their unique living characteristics, these engineered bacteria‐based living probes are able to actively target lesion sites, showing the potential to improve the precision of bioimaging and diagnosis in vivo in real time. In this section, we discuss the current strategies in the preparation of bacteria‐based living probes by bioengineering.

#### 2.1.1 Genetic Engineering

Genetic engineering has been a designable and easy‐to‐operate technology to achieve controllable and permanent production of functional agents in bacteria. Thus, it has become the most frequently‐used biotechnology in the study of bacteria‐mediated biomedical applications. Once the related genes encoding fluorescent proteins or luciferases are integrated into the bacterial genome or transformed into bacteria by genetic engineering, the obtained bacteria are able to produce corresponding proteins, and then can be employed as bacteria‐based living probes for in vivo biological imaging of the intestine, wound, tumor, and normal organs.^[^ ^20^, ^21^, ^22^, ^23^ ^]^

**Figure 3:**
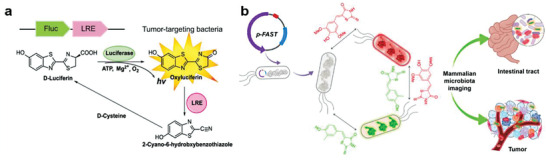
Preparation of bacteria‐based living probes by genetic engineering. a) Use of EcN expressing luciferase Fluc and luciferase regenerating enzyme LRE as living probes for bioluminescence imaging of tumors. Reproduced with permission.^[^ ^8^ ^]^ Copyright 2021, American Chemical Society. b) Smart bacteria‐based living probes obtained from FAST‐expressing EcN for dual fluorescence imaging of mammalian microbiota. Reproduced with permission.^[^ ^25^ ^]^ Copyright 2023, Elsevier.

Benefiting from the discovery of various imageable proteins and the advances of genetic engineering, plenty of engineered bacteria producing imageable signals have been constructed and provided foundation for their applications in bioimaging and diagnosis.^[^ ^4^, ^6^ ^]^ Tangney et al. designed several strains, such as *Bifidobacterium* and *Escherichia coli* (*E. coli*), to carry the *lux*ABCDE cassette via gene engineering for bioluminescence imaging.^[^ ^23^ ^]^ These bacteria‐based living probes could be used to co‐localize and track bacteria in mouse tumor models using three dimensional (3D) diffuse optical tomography and micro‐computed tomography (mCT). The growth of these luxs‐labeled bacteria could also be monitored in the tissues of mice. Zhang et al. constructed a probiotic strain *E. coli* Nissle 1917 (EcN) through genetic engineering by recombining and integrating the genes to express luciferase Fluc and luciferase regenerating enzyme LRE, generating robust, continuous, and red‐shifted bio‐luminescent tracking of bacteria both in vitro and in vivo. This work provides an optical platform for in vivo studies of bacteria‐mediated cancer therapy (**Figure** **3**a).^[^ ^8^ ^]^ Recently, Min et al. reported a repressor‐regulated tetracycline efflux system that combined a therapeutic gene (*cytolysin A*) and an imaging reporter gene (*renilla luciferase* variant 8) into the expression plasmid of attenuated *Salmonella typhimurium* (*S. typhimurium*).^[^ ^24^ ^]^ In addition to in vivo tracking, the engineered bacteria achieved precious localization to tumor site, significant inhibition to primary and metastatic tumors, and remarkable survival extension in tumor‐bearing mice.

Since the successful cloning and expression of green fluorescent protein (GFP) in organisms in the early 1990s, a serial of enhanced GFP derivatives and other fluorescent proteins have been constantly reported in different bacterial species.^[^ ^20^, ^21^ ^]^ For example, the *mCherry* gene, as the best‐known derivative of the red fluorescent proteins (RFPs), has been integrated and encoded into *E. coli*, achieving a shortened maturation time and improved brightness and photostability.^[^ ^22^ ^]^ There is another limitation of fluorescent proteins for in vivo application, namely the requirement of molecular oxygen for fluorescence emission. To address this challenge, Liu et al. encoded bacteria with a fluorescence‐activating and absorption‐shifting tag (FAST) to prepare smart living probes, which were successfully applied for real‐time dynamic, dual‐modal, and molecular oxygen‐independent imaging of the host microbiota (Figure 3b).^[^ ^25^ ^]^ By adding or removing specific fluorogens, the fluorescence of bacteria‐based FAST probe could be rapidly turned on/off on demand. As a type of endogenous fluorescent probe, FAST‐containing EcN could be used for anaerobically tracking both gut and tumor microbiota, and showed flexibility in on‐demand fluorescence turn‐on/off and reversibly switchable emission bands for intelligent and dual‐color imaging.

For better tissue penetration depth, other imageable signals have been also produced in bacteria for bioimaging. For example, Shapiro et al. optimized and cloned acoustic reporter genes (ARGs) into *E. coli* and *S. typhimurium* to express a unique class of gas‐filled protein nanostructures, which were successfully localized and non‐invasively imaged in the GI tract and tumor sites.^[^ ^26^ ^]^ Actually, the authors conducted massive screening work in different bacteria and archaea to obtain ARGs variations that have stronger ultrasonic contrast and then introduced the screened ARGs into engineered bacteria. Afterward, they found that the genetically engineered bacteria carrying improved ARGs presented much stronger non‐linear contrast than the first‐generation counterparts and could be applied for non‐invasive imaging to track the progressions of tumor gene expression and growth in a mouse model of breast cancer (Figure 3c).^[^ ^27^ ^]^ The expression of gas‐filled vesicle proteins provides a platform for deep‐tissue imaging and has great significance for the study of mammalian intestinal microbial activity and tumor imaging.

#### 2.1.2 Metabolic Labeling

Metabolic labeling refers to a metabolism‐based chemical biology strategy to label bacterial cells with chemically labeled signal molecules that can be internalized as nutrients for bacterial growth. Namely, bioimaging signal molecules can be automatically integrated into cellular biomolecules through endogenous biosynthetic process.^[^ ^28^ ^]^ Compared to genetic engineering, the preparation of bacteria‐based living probes by metabolic labeling is generally easier to operate. So far, the metabolic labeling can be simply divided into two types: supplementation of isotope‐/radioisotope‐labeled or fluorescent dye‐labeled metabolic substrates during bacterial proliferation for directly introducing signaling molecules via metabolic labeling; labeling with clickable alkyne‐/azide‐containing molecules through metabolic labeling for indirectly introducing signaling molecules through bioorthogonal conjugation.^[^ ^29^ ^]^ Correspondingly, these signal molecules can be detected by high‐resolution secondary ion mass spectrometry, PET, or optical imaging.^[^ ^9^, ^28^, ^30^ ^]^

**Figure 4:**
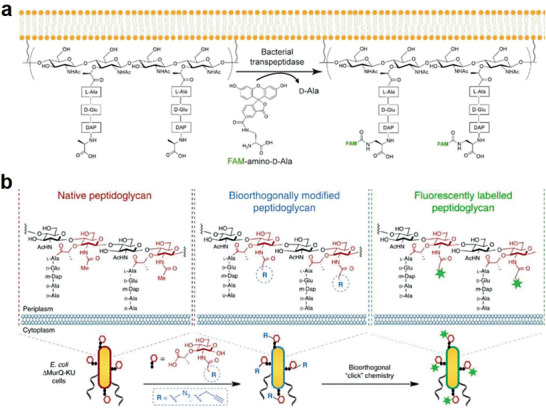
Preparation of bacteria‐based living probes by metabolic labeling. a) D‐amino acid‐mediated modification of gut bacteria to form in situ hybridization probes by direct metabolic labeling. Reproduced with permission.^[^ ^30^ ^]^ Copyright 2020, Wiley‐VCH.b) Indirectly metabolic labeling of *E*. *coli* for fluorescence imaging. Reproduced with permission.^[^ ^31^ ^]^ Copyright 2017, Springer Nature.

The direct labeling of isotopic signal molecules through bacterial metabolism has been extensively studied in the past years. Jain et al. used commercially available fluorine‐18 (^18^F) labeled fluorodeoxyglucose (^18^F‐FDG) to obtain 2‐deoxy‐2‐[^18^F] fluoro‐D‐sorbitol (^18^F‐FDS), which could integrate into bacterial cell wall directly and become a radioactive probe for *Enterobacteriaceae*.^[^ ^9^ ^]^ Interestingly, ^18^F‐FDS could not enrich in Gram‐positive bacteria or mammalian cells but selectively accumulate in *Enterobacteriaceae*. This specific labeling was further proved in vivo using a murine model of myositis. For example, when Gram‐positive *Staphylococcus aureus* (*S. aureus*) and Gram‐negative *E. coli* were mixed for thigh co‐infection, only the infection site caused by *E. coli* exhibited significant PET imaging signal. Moreover, in Klebsiella pneumonia and brain infection models, true infection could be rapidly distinguished from sterile inflammation by ^18^F‐FDS PET imaging and the infection sites could be specifically localized by providing a 3D holistic view of the animals. This work proves that ^18^F‐FDS can selectively accumulate in Gram‐negative bacteria *Enterobacteriaceae*, and be a candidate probe for future translation for clinical PET imaging. In addition, extensive studies have also focused on the introduction of fluorescent signal molecules through bacterial metabolic labeling. For example, Kasper et al. chose D‐amino acid (FDAA) derived fluorescent signal molecules that could be metabolized by bacteria, particularly in the gut, and intragastrically administered FDAA to mice to directly label the gut microbiota, providing a tool to visualize the gut microbiome.^[^ ^28^ ^]^ Recently, Yang and co‐workers proposed a metabolic D‐amino acid‐based labeling and in situ hybridization‐facilitated (MeDabLISH) strategy for quantitative analysis of the indigenous metabolic status of gut bacteria (**Figure** **4**a).^[^ ^30^ ^]^ In this work, they demonstrated that the fluorescence intensity of FDAA labeled bacteria was closely related to their temporal and steady‐state metabolic status. Moreover, the basal metabolic levels of intestinal bacterial genera in mice were analyzed by fluorescence in situ hybridization probes and the metabolic activities of different gut bacterial genera showed that Gram‐negative bacteria had stronger metabolic activity during the day, while Gram‐positive bacteria had higher metabolic activity at night in mouse gut microbiota.^[^ ^30^ ^]^

The indirect strategy of combining azide/alkyne‐containing amino acid or sugar metabolism and click chemistry for signal molecule conjugation on the surface of bacteria has also been widely used to prepare bacteria‐based living probes in recent years. Joshi et al. incorporated a non‐standard amino acid into bacteria and then incubated them with p‐azido‐L‐phenylalanine pAzF to express an azide group‐containing cell surface protein CsgA through metabolism.^[^ ^29^ ^]^ By a copper‐free click reaction, the engineered bacteria were constructed to be a fluorescent bacteria‐based probe by covalent labeling with dibenzocyclooctyl DBCO group‐conjugated Cy5 dye. Similarly, basing on the cell wall component of peptidoglycan (PG), Grimes's group introduced azide‐containing monosaccharide in the N‐acetyl‐muramic acid carbohydrate derivatives during metabolic cell wall recycling and biosynthetic process, with the fluorescent dyes conjugated onto the azide‐functionalized bacterial surface by click chemistry (Figure 4b).^[^ ^31^ ^]^ This work opens a window to see how the immune system recognizes bacteria and understand the construction of bacterial cell wall, providing essential information for designing new antibiotics.

### 2.2 Construction of Bacteria‐Based Living Probes by Chemical Modification

As the outermost protective layer of bacteria, cell wall consists of several compositions, mainly including peptidoglycan, teichoic acid, lipids, and proteins.^[^ ^32^ ^]^ These compositions are rich in a large number of functional groups, such as amino, carboxyl, phosphoric acid, and sulfhydryl groups.^[^ ^33^, ^34^ ^]^ Thus, the presence of these functional groups makes bacterial surface electronegative and chemically modifiable. In this part, chemical modification of bacterial surface through varied molecular structures and functional motifs by either non‐covalent interaction or covalent bonding was reviewed.

#### 2.2.1 Non‐Covalent Modification

Non‐covalent interactions are ubiquitous in biological systems, such as protein folding and DNA double helix structure.^[^ ^35^, ^36^ ^]^ It has been found that the structural compositions and electronegativity of bacterial surfaces provide great feasibility for their chemical modification through non‐covalent interactions, such as electrostatic interaction, metal coordination, and hydrophobic interaction.^[^ ^37^, ^38^, ^39^ ^]^ In recent years, various functional molecules and materials have been decorated on bacterial surface basing on non‐covalent interaction, emerging as a unique strategy to endow bacteria with specific exogenous functions for preparing bacteria‐based living probes.^[^ ^40^, ^41^ ^]^ For example, positively charged signal molecules can be electrostatically bound on bacterial surface by exploiting their mutual electronegativity. On the other hand, special structures on bacterial surface can also facilitate the binding of specific signal molecules and nanoparticles, such as conjugated polymers, aggregation‐induced emission (AIE) molecules, quantum dots, and fluorescent nanoparticles.

**Figure 5:**
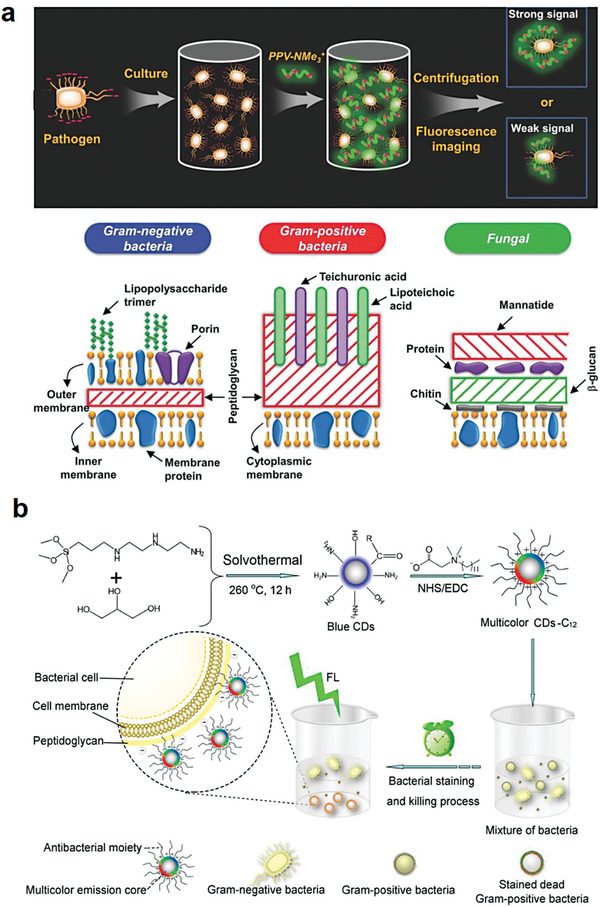
Preparation of bacteria‐based living probes by non‐covalently modification. a) Modifying bacteria with cationic polymer PPV‐NMe^3+^ via hydrophobic interaction for bacterial imaging. Reproduced with permission.^[^ ^10^ ^]^ Copyright 2014, Wiley‐VCH b) Decorating bacteria with multi‐color CDs via dual electrostatic and hydrophobic interaction for bacterial imaging. Reproduced with permission.^[^ ^48^ ^]^ Copyright 2016, American Chemical Society.

In 2011, Wang's group reported a multifunctional cationic poly (p‐phenylene vinylene) derivative bearing polyethylene glycol (PEG) side chains (PPV‐1), which exhibited self‐luminous properties and could selectively identify bacteria through electrostatic interaction, and be used for specific recognition, imaging, and killing of bacteria instead of mammalian cells without additional labeling steps.^[^ ^37^ ^]^ Later, they synthesized a cationic poly (p‐phenylene vinylene) (PPV‐NMe_3_^+^), which could bind to *Streptococcus albicans* and *E. coli* mainly by electrostatic interaction, and bind to *Bacillus subtilis* majorly basing on hydrophobic interaction (**Figure** **5**a).^[^ ^10^ ^]^ By changing the ionic concentration of the buffer solution, this polymer could be consequently applied to identify both fungi and bacteria in a quick yet easy manner via bioimaging. For example, Yan et al. synthesized a polymer (PLNP@PANI‐GCS) by grafting polyaniline (PANI) and ethylene glycol chitosan (GCS) onto the surface of persistent luminescent nanoparticles (PLNPs).^[^ ^42^ ^]^ Taking advantage of the surface charge transition of GCS in PLNPs in response to environmental pH, PLNP@PANI‐GCS could electrostatically bind to bacterial surface by adjusting the pH value. Moreover, using the long‐term sustained luminescence of PLNPs and the pH‐dependent photothermal conversion property of polyaniline, continuous fluorescence imaging could be achieved via tuning pH value.^[^ ^42^ ^]^

AIE molecules, with strong photo stability and low toxicity, can also be non‐covalently modified on bacterial surface for preparing bacteria‐based living probes.^[^ ^39^, ^43^, ^44^ ^]^ For example, Tang's group reported an AIE fluorogen, TPE‐Bac, containing two long alkyl chains and positively charged amine groups, which could bind to bacteria by electrostatic interaction and be used for bacterial imaging without involvement of washing procedure.^[^ ^38^ ^]^ Specifically, this molecular structure could intercalate into bacterial membrane and destroy the membrane integrity subsequently. Moreover, TPE‐Bac could be used as a photosensitizer to generate reactive oxygen species (ROS), which effectively deactivates both Gram‐positive and Gram‐negative bacteria. Zhou's group synthesized a series of aldehyde, carboxylic acid, and quaternary ammonium salt‐functionalized tetraphenylethylenes, which were able to aggregate on the bacterial surface through dual electrostatic and hydrophobic interactions.^[^ ^45^ ^]^ By employing these fluorescent materials, they identified eight representative bacteria by simply detecting fluorescence intensity, suggesting an alternative for bacterial bioimaging. Similarly, Sarma's group reported that the intrinsic positively‐charged *C*_3_‐symmertric molecules were self‐aggregated on bacterial surface through electrostatic interaction and simultaneously applied in bacterial detection, imaging, and killing.^[^ ^46^ ^]^ This work proposes a feasible way to design multifunctional broad‐spectrum antimicrobial systems for rapid and real‐time detection and wash‐free imaging of drug‐resistant microbes.

Because of their good optical property, low cost, and excellent biocompatibility, a kind of new fluorescent nanomaterials, carbon quantum dots, have been explored as bioimaging signal molecules for developing bacteria‐based living probes.^[^ ^47^ ^]^ Wu's group synthesized quaternized carbon dots (CDs) for bacterial surface decoration using a simple carboxyl‐amine reaction via dual electrostatic and hydrophobic interaction. The obtained bacteria‐based living probes showed superior capability for super‐resolution imaging of bacteria due to the polarity‐sensitive fluorescence emission property of quaternized CDs (Figure 5b).^[^ ^48^ ^]^

In addition to the conjugated polymers, AIE molecules, quantum dots, other specific signal molecules, and nanoparticles have also been reported for preparing bacteria‐based living probes by non‐covalent interactions. As well known, vancomycin can selectively bind to the D‐Ala‐D‐Ala moiety on the surface of Gram‐positive bacteria, while L‐tryptophan can target the peptidoglycan in the bacterial cell wall of both Gram‐positive and Gram‐negative bacteria.^[^ ^49^ ^]^ Wang et al. prepared a new photoacoustic contrast agent, which was composed of pyropheophorbide‐α as a signaling molecule, Pro‐Leu‐Gly‐Val‐Arg‐Gly as an enzyme‐reactive peptide linker, and vancomycin as a targeting ligand. This photoacoustic contrast agent could be utilized for specific and sensitive imaging of bacterial infection in vivo.^[^ ^39^ ^]^ Moreover, He et al. synthesized a novel bacteria‐based probe by modifying fluorescent silicon nanoparticles with vancomycin on the surface (SiNPs‐Van), which demonstrated great selectivity for Gram‐positive bacterial infection, favorable fluorescence efficiency, good biocompatibility, and strong resistance to photobleaching.^[^ ^50^ ^]^

#### 2.2.2 Covalent Conjugation

Thanks to the presence of reactive carboxyl, amino, hydroxyl, and sulfhydryl groups, different functional molecules and nanoparticles can be covalently anchored on bacterial surface by chemical reactions. Compared to non‐covalent modification, covalent conjugation usually enables more stable immobilization of signal molecules on bacterial surface. To make full use of the reactive groups on bacterial surface, Liu et al. employed a simple one‐step imidoester reaction for converting primary amine groups on bacterial surface to free thiols, which exhibited improved efficiency in attaching signal molecules by click chemistry. The gained bacteria‐based living probes could be applied for bioimaging in different mucus interfaces of the host.^[^ ^51^ ^]^ They also decorated bacteria with tumor‐targeting aptamer (AS1411) on the surface through amide condensation reaction and tracked the localization of modified bacteria in tumor site after systemic injection by employing the fluorescence signal of fluorochrome Cyanine5 (Cy5) attached at 5′ end of the AS1411 sequence.^[^ ^52^ ^]^ Xie et al. designed a relebactam‐derived fluorogenic reagent to covalently label serine *β*‐lactamases, the major cause of bacterial resistance to *β*‐lactam antibiotics. This probe exhibited a high selectivity, which could produce a strong near‐infrared (NIR) fluorescence signal after covalently binding to SBLs, thereby allowing wash‐free visualization of antimicrobial‐resistant bacteria.^[^ ^53^ ^]^

**Figure 6:**
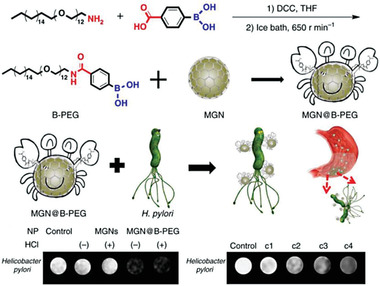
Preparation of bacteria‐based living probes by covalent modification. Attachment of MGNs onto bacteria via a covalent bond. Reproduced with permission.^[^ ^54^ ^]^ Copyright 2017, Springer Nature.

In addition, various functional nanoparticles can be covalently adopted onto bacterial surface. For instance, Cai et al. prepared a type of indocyanine green‐loaded nanophotosensitizers for covalent attachment onto the surface of YB1 bacteria by an amide bond (YB1‐INPs) without impacting bacterial activity.^[^ ^11^ ^]^ The obtained YB1‐INPs bacteria showed specific hypoxia targeting solid tumors, satisfactory photothermal conversion, and efficient fluorescence imaging. Besides, Tan's group reported the use of magnetic graphite nanocapsules (MGNs) to modify bacterial surface through the formation of a reversible dynamic boronate bond between boron polyethylene glycol chains and bacterial peptidoglycan. Interestingly, the prepared bacteria could be employed for targeted MRI detection of *Helicobacter pylori* (*H. pylori*) (**Figure** **6**).^[^ ^54^ ^]^ This work suggests an elegant approach to prepare bacteria‐based MRI imaging probes and the MGNs‐mediated enhanced MRI can be a promising platform for deep‐tissue bioimaging and diagnosis.

### 2.3 Construction of Bacteria‐Based Living Probes by Intracellular Loading

**Figure 7:**
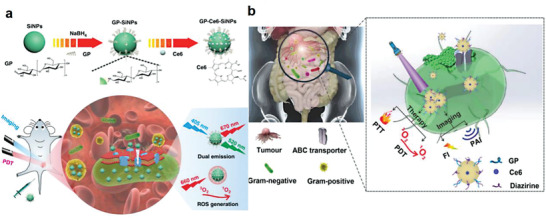
Preparation of bacteria‐based living probes by intracellular loading. a) Design of multifunctional nanoagents for bacteria‐based imaging probes via intracellular loading. Reproduced with permission.^[^ ^12^ ^]^ Copyright 2019, Springer Nature. b) Bacterial uptake of gold nanoparticles for intracellular aggregation‐enhanced imaging. Reproduced with permission.^[^ ^59^ ^]^ Copyright 2022, Springer Nature.

Uptake of macromolecules and granular substances is a common physiological phenomenon in both eukaryotic cells and prokaryotic cells. Currently, relying on their high efficiency to uptake nanoparticles, various organic and inorganic luminescent nanoprobes with fluorescent, NIR, photoacoustic, and MRI properties have been loaded into mammalian cells for intracellular imaging. Although the cell internalization mechanism is different from mammalian cells, bacteria are able to uptake nanoparticles.^[^ ^55^, ^56^, ^57^, ^58^ ^]^ Thus, by virtue of this merit, cell uptake‐based intracellular loading of bioimaging nanoparticles can be exploited as a facile approach to develop bacteria‐based living probes. It is worth mentioning that large‐sized nanoparticles are generally difficult to enter bacterial cells due to their limited size, ≈0.5–5 µm, as well as the existence of bacterial membranes and dense cell walls. On the contrary, the small‐sized nanoparticles can easily accumulate in bacterial cells for efficient imaging. For example, He's group synthesized glucose poly[4‐O‐(α‐D‐Glucopyranosyl)‐D‐glucopyranose] modified and chlorin e6‐encapsulated fluorescent silicon nanoparticles (SiNPs), which were used to prepare bacterial living probes via intracellular loading (**Figure** **7**a).^[^ ^12^ ^]^ As demonstrated, the resulting nanoagents could be rapidly internalized into both Gram‐negative and Gram‐positive bacteria through an ATP‐binding cassette (ABC) transporter pathway and used for imaging of bacterial infections. Subsequently, they prepared glucose polymer‐modified gold nanoparticles that could be actively internalized by different bacterial species and remarkably aggregated within bacterial cells under laser irradiation, resulting in higher photoacoustic signals than those of non‐aggregated counterparts (Figure 7b).^[^ ^59^ ^]^ This work enables the visualization of a variety of bacteria by intracellular loading of nanoprobes, taking an important step forward in the study of microbial ecosystem. More recently, the ABC sugar transporter has been used for selective delivery of luciferase and luciferin to different natural bacteria by hitchhiking to α‐(1‐4)‐glucosidcally linked glucose polymer‐linked nanoparticles, achieving ex vivo bioluminescence imaging of human vitreous containing ten pathogens that were collected from patients with bacterial endophthalmitis. Furthermore, this platform exhibited the capability for sensitive imaging and photothermal therapy in deep tissues, as bacterial or non‐bacterial nephritis and colitis in mice could be perfectly differentiated, which were difficult to be distinguished by chemiluminescent counterparts.^[^ ^60^ ^]^

### 2.4 Construction of Bacteria‐Based Living Probes Basing on Optical Manipulation

Optical manipulation, as a gentle and non‐mechanical contact method that can accurately capture and manipulate inorganic or organic nanomaterials, living cells, viral particles, etc. through light beams, has promoted rapid development in many fields including physics, biochemistry, and biomedicine.^[^ ^61^, ^62^, ^63^ ^]^ Objects ranging in size from nanometres to microns can be captured and moved by a strongly focused beam, achieving precise alignment. In recent years, this non‐invasive and contactless method has been adopted to prepare bacteria‐based living probes, which can be used for optical detection and diagnosis of various diseases.^[^ ^64^, ^65^, ^66^, ^67^ ^]^ For example, Li and colleagues prepared biophotonic waveguides (bio‐WGs) with *E. coli* through an optical approach, which had both different lengths and good light propagation performance and could detect the propagation signal in real time.^[^ ^68^ ^]^ This strategy provides a seamless interface between the optical and biological worlds with natural materials and opens up new opportunities for direct sensing and detection of biological signals and information in a biocompatible microenvironment. Subsequently, they optically assembled *E. coli* and chlorella cells together to form 1D periodic cell structure with controllable configurations and lengths, resulting in the controllable mapping of different cells.^[^ ^69^ ^]^ In addition, the authors assembled *E. coli* into structures with different branches and lengths by optical manipulation and investigated optical propagation performance of these branches and the robustness of the structures.^[^ ^70^ ^]^ The results showed that the bacterial branching structure can be used as a multidirectional waveguide and beam splitter to connect the biological and optical worlds. They also used individual upconversion nanoparticles for single bacterial labeling by this optical strategy, providing an alternative for labeling, detection, and real‐time analysis of single‐pathogenic‐bacterium at single particle and single cell level.^[^ ^71^ ^]^

## 3 Types of Bacteria‐Based Living Probes

**Figure 8:**
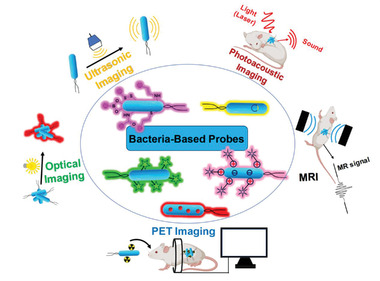
Types of bacteria‐based living probes for different imaging modalities, including optical imaging, ultrasonic imaging, photoacoustic imaging, MRI, and PET imaging.

Non‐invasive imaging techniques are appealing tools in the arsenal of clinical diagnostics. A diverse range of imaging modalities are now available, from techniques that enable entire organism anatomical imaging (such as MRI) to others that provide specific molecular imaging (such as PET). In the past decades, several nanoparticle‐based contrast agents have been developed to overcome issues that plague conventional contrast agents.^[^ ^72^ ^]^ Differently, as the main components of the mammalian microbiomes, both Gram‐positive and Gram‐negative bacteria can colonize different biological interfaces in vivo, and affect human health and even the process of diseases. In this section, multifunctional bacteria‐based living probes derived from engineered bacteria were reviewed basing on optical imaging, ultrasonic imaging, photoacoustic imaging, MRI, and PET imaging (**Figure** **8**).

### 3.1 Bacteria‐Based Living Probes for Optical Imaging

Optical imaging, mainly including fluorescence imaging and bioluminescence imaging, is a visual imaging technology by using the luminous intensity of probes as a detection signal and has attracted increasing attention for both preclinical and clinical applications.^[^ ^73^ ^]^ Optical imaging is highly efficient and sensitive, with clear advantages, such as non‐invasion, real‐time imaging, high resolution, absence of radiation‐related risks, and relatively low costs.^[^ ^73^ ^]^ Bacteria‐based living probes that can be used for in vivo real‐time observation are presented for both bioluminescence imaging and fluorescence imaging.

#### 3.1.1 Bacteria‐Based Living Probes for Bioluminescence Imaging

Bioluminescence imaging is a non‐invasive optical imaging technology based on enzymatic reaction of luciferase and luciferin in living organisms, which belongs to a natural phenomenon and does not require light excitation compared to fluorescence imaging.^[^ ^74^ ^]^ Thanks to its high sensitivity, resolution, and selectivity, bioluminescence imaging has been widely applied in the visualization and monitoring of complicated molecular and cellular processes, and the labeling of eukaryotic cells and prokaryotic cells.^[^ ^75^, ^76^ ^]^

**Figure 9:**
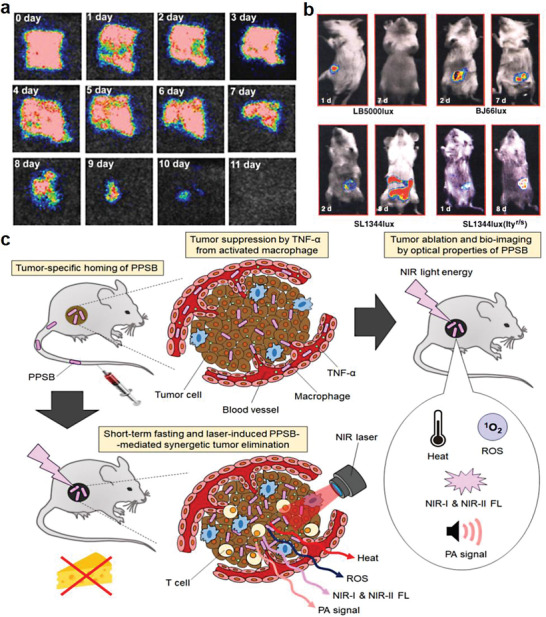
Delivery of bacteria‐based living probes for optical imaging. (a and b) Bioluminescence imaging. Reproduced with permission.^[^ ^79^ ^]^ Copyright 2013, Wiley‐VCH. Reproduced with permission.^[^ ^82^ ^]^ Copyright 1995, Wiley‐VCH. c) NIR imaging at 650–900 nm. Reproduced with permission.^[^ ^90^ ^]^ Copyright 2021, Elsevier.

Different from bioluminescence systems in mammalian cells, which require the supply of exogenous substrates to produce bioluminescence, bacterial bioluminescence systems, constructed by bacterial luciferase gene cassette that encodes all the proteins required for bioluminescence, including bacterial luciferase, substrate, and substrate‐regenerating enzymes, are fully autonomous, substrate‐free, and available in both prokaryotic and eukaryotic hosts.^[^ ^77^, ^78^ ^]^ Therefore, the types of bacterial strains including *E. coli*, *S. typhimurium*, *Bifidobacterium longum*, *Mycobacterium ulcerans* (*M. ulcerans*), and *Clostridium novyi* (*C. novyi*), which have natural advantages to selectively colonize the hypoxic area of tumor for survival and reproduction, have been labeled with bioluminescence and used as feasible probes to track bacteria and diagnose diseases in vivo (**Figure** **9**a).^[^ ^79^, ^80^, ^81^, ^82^ ^]^

In terms of Gram‐negative bacteria, Min et al. developed a plasmid containing *lux*CDABE from *Photobacterium leiognathi* (*P. leiognathi*) and transformed it into *E. coli* for tumor and metastase imaging in mice via intravenous injection.^[^ ^81^ ^]^ Contag et al. constructed a vector with constitutive expression of bacterial luciferase for bioluminescent labeling of *S. typhimurium*, allowing for accurate real‐time tracking of bacterial infection in mice (Figure 9b).^[^ ^82^ ^]^ As the *lux*CDABE operon from *P. leiognathi* is incompatible in Gram‐positive bacteria, the cassette has to be recognized to *lux*ABCDE instead.^[^ ^74^ ^]^ For example, Moon et al. prepared a bioluminescent *Lactobacillus plantarum* (*L. plantarum*) strain with a P32 promoter from the pMG36e lactococcal expression vector and *lux*ABCDE operon for non‐invasive in vivo tracking in the intestinal tract of mice.^[^ ^83^ ^]^ Similarly, Zhang et al. created a recombinant bioluminescent strain of *M. ulcerans* carrying *luxAB* from *Vibrio harveyi* for rapid, serial, non‐invasive, and real‐time evaluation of antimicrobial therapeutic efficacy in vivo.^[^ ^83^, ^84^, ^85^ ^]^

#### 3.1.2 Bacteria‐Based Living Probes for Fluorescence Imaging

Fluorescence imaging is another attractive optical imaging to observe and monitor the labeled organisms with the assistance of fluorescent probes in a non‐invasive and real‐time way. Currently, fluorescence imaging has been developed rapidly and is used widely in life science, surgery guiding, and medicine. There are a wide variety of probes including fluorescent proteins, fluorescent dyes, quantum dots, and others, which can be introduced in or on bacteria by genetic engineering or surface modification to prepare bacteria‐based living probes.^[^ ^73^ ^]^

Several studies have described the imaging of bacteria (such as *E. coli* and *S. typhimurium*) by heterologously expressing either oxygen‐dependent fluorescent proteins, such as GFP, yellow fluorescent protein (YFP), and RFP, or oxygen‐independent fluorescent proteins (for example, FAST) via genetic engineering.

To expand the methods of preparing bacterial fluorescent probes, various chemical labeling strategies have been developed.^[^ ^85^, ^86^ ^]^ Compared to traditional genetic engineering, chemical labeling is simpler and more flexible, especially for labeling anaerobic bacteria with oxygen‐independent fluorescent dyes. For example, to examine the colonization of *Bacteroides fragilis* (*B. fragilis*) (ZY‐312) in the GI tract, Xu et al. prepared bacterial probes basing on AF647‐dibenzocyclooctyne (DIBO) labeling by using metabolic oligosaccharide engineering and bioorthogonal click chemistry (BCC).^[^ ^86^ ^]^ The biomolecule of the target strain was modified with a small reactive group through a cell's endogenous biosynthesis function and then reacted with the second functional group through BCC to form a stable covalent bond. In this work, they incubated bacteria with AF647‐DIBO in dark for 5 h to obtain the optimized bacterial probes, indicating that the process of labeling was simple and convenient. Notably, this strategy has been widely applied in various types of living bacteria to study glycoconjugates and polysaccharides, such as *S. aureus* and *Bacteroides ovatus*.^[^ ^86^ ^]^ In addition, inspired by the antibacterial mechanism of antibiotics, a variety of bacterial probes have been designed and prepared. Vancomycin, which has been used clinically for over 60 years, is a glycopeptide antibiotic that specifically targets Gram‐positive bacteria and inhibits cell wall synthesis. Relying on its Gram‐positive bacteria‐targeting property, different bacterial probes have been developed basing on labeled vancomycin. Dam et al. used fluorophore‐conjugated vancomycin as a fluorescence imaging probe, which was successfully applied as a diagnostic tool to detect bacterial infection both in vitro and in vivo.^[^ ^73^ ^]^ This type of fluorescent probes could maintain both fluorescence characteristics and the ability of vancomycin to bind bacterial cell wall, which was crucial for its function as a probe.

#### 3.1.3 Bacteria‐Based Living Probes for NIR Imaging

Despite its appealing characteristic, fluorescence imaging may suffer limitations for detecting deep tissues, as conventional fluorescent signals have relatively weak penetrability. To tackle these limitations, NIR fluorescent agents with absorption wavelengths from 650 to 900 nm have been developed.^[^ ^87^, ^88^ ^]^ Comparing to traditional fluorescent agents, NIR fluorescent agents achieve a higher signal‐to‐noise ratio and stronger tissue penetration, resulting in enhanced performance in deep‐tissue imaging, since biological tissues have weak light absorption at NIR region and the attenuation length of NIR light is longer than that of visible light. In addition, the phototoxic effects are significantly reduced within NIR optical windows from 650 to 900 nm even at high irradiation intensities. These unique properties render NIR fluorescent probes attractive for living cell microscopy, especially for living cell diffraction‐unlimited super‐resolution (nanoscopy) applications that typically require higher irradiation doses.

To improve the quality of bioimaging in deeper tissues, different types of NIR fluorescent probes have been developed. Firstly, to detect bacteria in vivo, NIR fluorophores have been conjugated with antibiotics. As shown in Dam's work, a NIR dye of IRDye 800CW was combined with vancomycin and then used as an optical imaging agent to selectively target Gram‐positive bacteria in vivo.^[^ ^73^ ^]^ To monitor tumor in vivo in a fashion of spatiotemporally‐controllable and highly specific, Min et al. discovered a novel tumor‐targeting bacterium, *Rhodobacter sphaeroides* 2.4.1 (*R. sphaeroides*), a Gram‐negative purple bacterium in a rod shape, which could emit NIR fluorescence naturally near 900 nm.^[^ ^89^ ^]^ As demonstrated by in vivo imaging of different types of tumors, the NIR fluorescence signals from *R. sphaeroides* were much more pronounced and stronger in deep tissues than those from GFP and RFP expressing bacteria. Similarly, Miyako's group explored a novel bacteria‐based probe for NIR imaging.^[^ ^90^ ^]^ In this work, they discovered non‐pathogenic purple photosynthetic bacteria (PPSB) including *Rhodopseudomonas palustris* and *Blastochloris viridis*, which were multifunctional and biocompatible in cancer treatment (Figure 9c). As cancer theranostic probes, PPSB exhibited strong NIR‐I‐to‐NIR‐II reporter fluorescence (excitation from 785 to 850 nm) by using NIR‐I‐to‐NIR‐II biooptical window lights.

### 3.2 Bacteria‐Based Living Probes for Ultrasonic Imaging

Ultrasonic imaging is a common, inexpensive, and fundamental way for both biological visualization of molecular/cellular processes and clinically diagnosis/therapy of diseases. This imaging technology is mainly based on ultrasound, which can propagate deeply into living organisms with highly spatial and temporal resolution without coherence losing. ^[^ ^91^ ^]^

Many microorganisms including bacteria and planktonic algae have been found to produce intracellular air‐filled gas vesicles in varied shapes naturally and can be used as ultrasonic imaging probes.^[^ ^26^, ^92^ ^]^ Such gas vesicles are nanostructural with a size of ≈200 nm and composed of all‐protein shells those are encoded by ARGs. However, gas vesicles from different species have varied characteristics, including size, shape, echogenicity, and acoustic scattering, resulting in different performances in imaging applications.^[^ ^93^, ^94^, ^95^ ^]^ With the development of genetic technologies, ARG has been intensively optimized and introduced into bacteria and mammalian cells for monitoring their locations and functions in host organisms.^[^ ^27^, ^92^, ^96^, ^97^ ^]^ For example, to construct a bacterial probe with robust ultrasound contrast for ultrasonic imaging, Shapiro's group combined the structural *GvpA* and *GvpC* genes from cyanobacterium *Anabaena flos‐aquae* with the accessory genes *GvpR*‐*GvpU* from *Bacillus megaterium*, and transformed them into *E. coli* to produce hybrid gas vesicles with better performance for ultrasound imaging.^[^ ^26^ ^]^ Due to their substantially larger dimensions, a greater fraction of intracellular volume, and stronger ultrasound contrast, different ARG‐expressing bacteria have been engineered and used for in vivo microbial imaging. EcN was modified to form intracellular gas vesicles and shown distinguishable ultrasound signals in the GI tract at a concentration of 1 × 10^9^ cells per milliliter. Another example was the pathogenic bacterium *S. typhimurium*, which can invade and colonize tumor sites.^[^ ^98^, ^99^, ^100^ ^]^ Attenuated *S. typhimurium strain* ELH1301 carrying gas vesicles was able to be imaged non‐invasively by ultrasound after injection into tumors.^[^ ^92^ ^]^ In this work, the authors enabled genetically‐engineered bacteria probes visible in a non‐invasive manner in deep tissues and demonstrated the advantages of ultrasonic imaging in terms of spatial localization within deep organs inside mammalian hosts. As proved, such probes presented great potential to facilitate the studies of mammalian microbiome and the development of diagnostic and therapeutic agents.

### 3.3 Bacteria‐Based Living Probes for Photoacoustic Imaging

Photoacoustic imaging, termed optoacoustic imaging as well, is a new non‐invasive and non‐ionizing biomedical imaging modality, which is based on photoacoustic signals. Photoacoustic imaging combines the high selectivity of optical imaging and the deep penetration of ultrasound imaging, resulting in highly spatial and temporal resolution and spectrally‐enriched contrast. As its ability to avoid the influence of light scattering in principle, photoacoustic imaging can break through the “soft limit” of depth in high‐resolution optical imaging (1 mm), and achieve in vivo imaging of deep tissues (50 mm).^[^ ^101^ ^]^ Moreover, photoacoustic imaging can resolve oxygenated/deoxygenated haemoglobin, melanin, lipids, collagen, and sense multi‐wavelength photoacoustic signals in a label‐free way.^[^ ^102^ ^]^ Recently, various reporters or probes have been discovered and applied for photoacoustic imaging, including β‐galactosidase/X‐gal (5‐bromo‐4‐chloro‐3‐indolyl‐β‐D‐galactoside),^[^ ^103^ ^]^ phytochrome‐based infrared fluorescent proteins (iRFPs),^[^ ^103^, ^104^, ^105^ ^]^ and the types of pigments.^[^ ^106^, ^107^ ^]^

**Figure 10:**
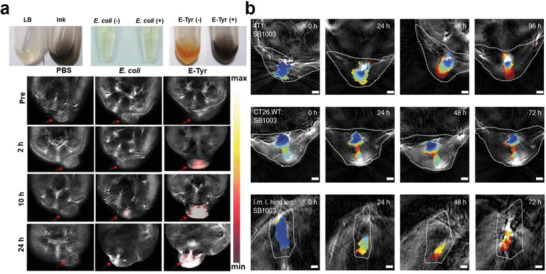
Delivery of bacteria‐based living probes for photoacoustic imaging. a) Construction of tyrosinase‐expressing *E. coli* and preparation of melanin bearing bacterial probe for photoacoustic imaging of CT26 tumors in mice. Reproduced with permission. ^[^ ^113^ ^]^ Copyright 2021, Springer Nature. b) BChl *a* expressing purple bacteria as living probes for photoacoustic imaging in different types of tumors in vivo. Reproduced with permission.^[^ ^114^ ^]^ Copyright 2019, Springer Nature.

LacZ, one of the well‐known chromogenic reporter genes, encodes an enzyme β‐galactosidase to metabolize optically transparent lactose‐like substrate X‐gal into 5,5′‐dibromo‐4,4′‐dichloro‐indigo, which is a stable insoluble blue product with a strong absorption in the red region of the optical spectrum from 605 to 665 nm.^[^ ^103^ ^]^ Li et al. genetically tagged tumors in rats for the first time by expressing β‐galactosidase and clearly visualized the tumors after injection of X‐gal by photoacoustic imaging under far‐red optical illumination, achieving a spatial resolution of <400 µm and a sensitivity of 0.5 µm.^[^ ^103^ ^]^ Similar to LacZ, iRFPs have been used in photoacoustic imaging by enzymatic reactions between bacterial phytochrome photoreceptors (BphPs) and biliverdin chromophore, providing significantly higher optoacoustic contrast than conventional fluorescence proteins. Shu et al. engineered a bacteriophytochrome from *Deinococcus radiodurans* and tagged the mammalian cells in mice with iRFP.^[^ ^88^ ^]^ With the incorporation of biliverdin as the chromophore, such iRFP exhibited maxima excitation and emission of 648 and 708 nm, respectively. Later, improved iRFPs without requirement of an external chromophore biliverdin addition have been reported. They showed higher effective brightness, intracellular stability, and greater photostability,^[^ ^108^ ^]^ such as IFP1.4,^[^ ^109^ ^]^ iRFP,^[^ ^108^ ^]^ and BphP1,^[^ ^110^ ^]^ which were demonstrated in mice. In contract to LacZ and traditional iRFPs, the types of pigments do not require injection of an extrinsic substrate for photoacoustic imaging. Currently, plenty of probes have been constructed for photoacoustic imaging, basing on these pigments. For example, tyrosinase can produce melanin, a dark and strongly absorbing pigment that is detectable using optoacoustic imaging systems. In addition to being formed into various nanoparticles for photoacoustic imaging, such as croconaine‐based nanoparticles,^[^ ^107^ ^]^ gold nanorods‐melanin nanohybrids^[^ ^111^ ^]^ and biopolymer‐melanin encapsulating OMVs,^[^ ^112^ ^]^ such pigments have also been extensively produced by bacteria to be used as living photoacoustic imaging tools for in vivo infection and tumor studies. Yun et al. genetically modified *E. coli* to express tyrosinase, which could use L‐tyrosine as the substrate to produce melanin and produce strong optoacoustic signals in the tumor microenvironment. Non‐invasive, longitudinal monitoring of bacterial targeting and proliferation in tumor could also be achieved by using this engineered *E. coli* (**Figure** **10**a).^[^ ^113^ ^]^ Another deep violet chromophore violacein, which has a strong absorption peak ≈590 nm and substantial absorbance above 650 nm and high photobleaching resistance, has also been heterogeneously expressed in *E. coli*. This type of *E. coli* can be differentiated well by multispectral photoacoustic imaging in strongly vascularized xenografted tumors in mice.^[^ ^12^ ^]^ Moreover, bacteria with naturally producing pigments have been found to have photoacoustic imaging performance. For example, purple bacteria rich in bacteriochlorophyll *a* (BChl *a*) and with strong photoacoustic signals beyond 800 nm have been utilized as probes for photoacoustic imaging (Figure 10b). Depending on the changes of their distinct spectral signatures, such bacterial probes could reveal massive information, for example, the heterogeneity of the tumor microenvironment.^[^ ^114^ ^]^

### 3.4 Bacteria‐Based Living Probes for MRI

MRI is playing an increasingly important role in our modern life, as it is non‐invasive and has been widely used as the most prominent medical imaging modality for high‐quality soft tissue imaging.^[^ ^115^ ^]^ Different from optical imaging and ultrasonic imaging, MRI is mainly based on the hydrogen content of organs in human body to distinguish corresponding organs and determine the condition of organ lesions.^[^ ^116^ ^]^ Thanks to the potential advantages, particularly high spatial resolution and synchronous acquisition of imaging signals without the requirement for ionizing radiation, MRI has been applied to monitor the changes of neural activity in the brain, detect early cancerous cells, and image microscopic biological structures.^[^ ^117^, ^118^, ^119^ ^]^

To further improve imaging sensitivity and efficiency of MRI, various probes that are composed of metals and metal oxides and able to enhance proton relaxation in targeted tissues have been developed, including synthetic magnetic nanoparticles (MNPs) by sophisticated chemical modifications,^[^ ^120^, ^121^ ^]^ metalloprotein‐based contrast agents by protein‐metal interactions,^[^ ^122^ ^]^ and bacteria and bacterial derivatives‐based probes by both surface decoration and genetic modification.^[^ ^123^ ^]^ Due to their excellent biomedical capabilities, including a high proportion of surface atoms, biodegradability, biocompatibility, safety, chemical stability, and rapid response, magnetic iron oxide nanoparticles consisting of maghemite (γ‐Fe_2_O_3_) or magnetite (Fe_3_O_4_) have been one of the most common MNPs for MRI, which can be either super paramagnetic or ferromagnetic.^[^ ^124^ ^]^

**Figure 11:**
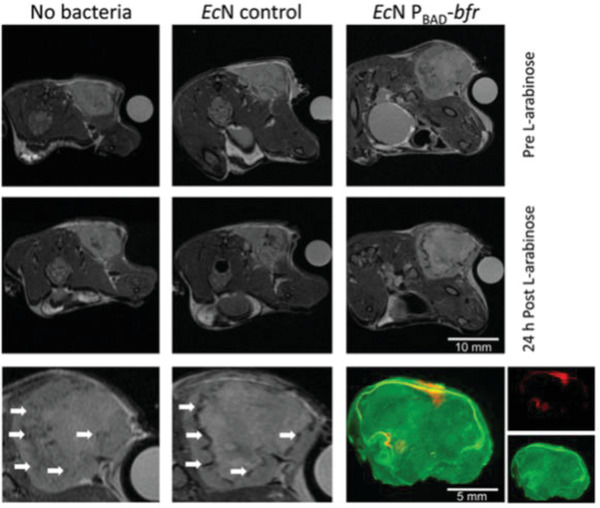
Delivery of bacteria‐based living probes for MRI. Bacterioferritin‐expressing EcN as living probes for MRI in murine tumors. Reproduced with permission.^[^ ^129^ ^]^ Copyright 2011, Public Library of Science.

Apart from synthetic magnetic probes for MRI, there are some special bacteria that either inherently produce MRI agents or acting as living MRI probes in a magical nature.^[^ ^125^ ^]^ For example, magnetotactic bacteria own magnetosome genomic islands and produce bacterial magnetosomes, mainly containing ferromagnetic and isostructural minerals (Fe_3_O_4_ or Fe_3_S_4_). Different from chemically synthetic magnetic nanoparticles, such bacterial magnetosomes have a biological lipoprotein membrane covered on the surface, exhibiting better biocompatibility and biosafety.^[^ ^126^ ^]^ In addition, such bacterial magnetosomes are modifiable via both chemical and biological strategies for multi‐functionalization, such as the combination of tumor specific targeting ability with enhanced permeability and retention.^[^ ^123^, ^127^ ^]^ Because of their magnetotaxis, aerotaxy, uniform shape, and size, as well as the exogenous functions, bacterial magnetosomes can target specific tissues with higher sensitivity and efficiency. Furthermore, since bacteria are born with the capabilities of targeting and colonizing lesion sites, they have been considered remarkably potential therapeutic agents and drug carriers. For example, *Magnetospirillum magneticum* AMB‐1 has been found to produce magnetite particles (Fe_3_O_4_) naturally and be able to accumulate and colonize tumors after both intravenous and intratumoral injection, acting as living probes for enhanced MRI.^[^ ^128^ ^]^ On the other hand, with the development of synthetic biological techniques, MR reporter genes to encode ferritin, transferrin, and magnetite‐forming related proteins have been inserted into *E. coli* and successful to increase intrinsic iron uptake for MRI. Benefiting from various iron uptake systems, EcN is a potential candidate for iron acquisition. Moreover, EcN has been genetically engineered to over‐express different types of bacterioferritin for optimizing iron accumulation and exploring promising probes for MRI in tumor‐bearing mice (**Figure** **11**).^[^ ^129^ ^]^

### 3.5 Bacteria‐Based Living Probes for PET Imaging

Compared to MRI, PET imaging has fundamental differences in imaging principle, as PET is a type of nuclear medicine imaging, that relying on the differences of radiation from the injected radioactive nuclear elements between normal tissues and disease regions.^[^ ^130^ ^]^ Currently, PET imaging has been developed rapidly for preclinical research and is used widely in clinics for the diagnosis of various diseases, such as cancers and bacterial infections.^[^ ^131^, ^132^ ^]^

**Figure 12:**
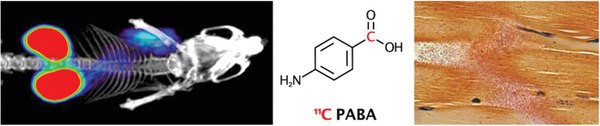
Delivery of bacteria‐based living probes for PET imaging. Metabolically labeled *E. coli* as living probes for [^11^C] PABA PET imaging in a murine myositis model. Reproduced with permission.^[^ ^138^ ^]^ Copyright 2018, American Chemical Society.

Currently, a variety of agents have been developed for PET imaging.^[^ ^130^, ^131^, ^132^ ^]^ For example, ^18^F‐FDG has been recognized as the best‐known and most frequently used radiotracer for PET imaging. Because ^18^F‐FDG is able to accumulate in metabolically active cells and phosphorylated to phosphorylated FDG by hexokinase, which cannot be further metabolized and consequently trapped inside the cells. Moreover, due to the high levels of glucose metabolism in tumor cells or inflammatory disease sites, FDG uptake is significantly enhanced than that in normal tissues. Therefore, ^18^F‐FDG has been broadly used in PET imaging for cancers.^[^ ^133^ ^]^ Moreover, ^18^F‐FDS has been reported to be preferentially absorbed by Gram‐negative bacteria, especially *Enterobacteriaceae*, allowing the diagnosis of Gram‐negative bacterial infections.^[^ ^134^, ^135^, ^136^ ^]^ Another radiolabeled compound, 2‐[^18^F] fluoro‐para‐aminobenzoic acid (2‐^18^F‐PABA) or [^11^C] PABA, has shown to be preferentially adsorbed and retained in both Gram‐positive *S. aureus* and Gram‐negative *E. coli* (**Figure** **12**).^[^ ^137^, ^138^ ^]^ Although there are numerous works by using radiolabeled agents to track and image bacteria in tumors or infected positions via metabolism in situ, there are few by using radiolabeled bacteria via surface decoration as probes for PET imaging, perhaps due to their short lifetimes.

## 4 Bacterial Derivatives‐Based Probes

Bacterial derivatives, including OMVs and spores, also present greatly promising potential as bioimaging probes. OMVs, which are double lipid layered vesicles in the size range of 20–250 nm in diameter, are released from bacteria. Due to the reservation of the natural characteristics of bacterial outer membranes and the same proteins, nucleic acids, and metabolites of parent cells, OMVs play essential roles in both intraspecific and interspecific communications between bacteria and surrounding environments.^[^ ^139^, ^140^ ^]^ Given their merits of unique physiological characteristics including loading capacity, nanosize, communications to bacteria and mammalian cells, and dual internal and external modifiability, OMVs serve as a versatile platform for a variety of bioimaging, such as optical imaging.^[^ ^141^, ^142^, ^143^ ^]^

**Figure 13:**
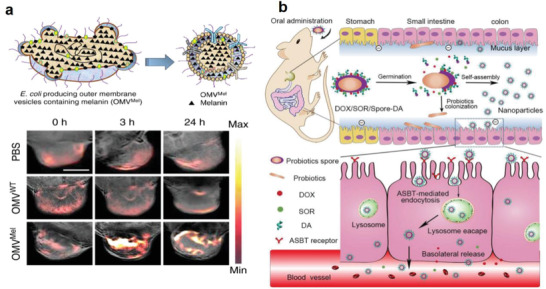
Bacterial derivative‐based probes for bioimaging. a) Generation of melanin carrying OMVs and the photoacoustic imaging in mouse tumor. Reproduced with permission.^[^ ^112^ ^]^ Copyright 2019, Springer Nature. b) Probiotic bacterium‐derived spores with drugs loaded on the surface for fluorescence imaging in the intestinal tract. Reproduced with permission.^[^ ^146^ ^]^ Copyright 2019, Wiley‐VCH.

For example, to explore the in vivo function of OMVs, Huang et al constructed the multifunctional OMVs from *E. coli* carrying nanoluciferase reporter for in vivo bioluminescence imaging.^[^ ^141^ ^]^ In this work, with adding the substrate of furimazine, these secreted OMVs with various functions could emit strong blue luminescence at 460 nm, which was used for bioluminescence‐based optical detection of immunoglobulin G and recognition of biomarkers on cancer cells. Moreover, to enable the distribution of OMVs released from gut microbes as well as their interactions with other gut microbes or mammalian host cells in the intestine visible and traceable, OMVs derived from FAST‐expressing EcN (OMVs‐FAST) were produced by inducing with antibiotic and isopropyl‐beta‐D‐thiogalactopyranoside (IPTG).^[^ ^142^ ^]^ As supported by both in vitro and in vivo data, by the merits of their natural structure and molecular oxygen‐independent emission, OMVs‐FAST exhibited great potential to be an intelligent endogenous fluorescent probe for tracking gut microbiota‐derived OMVs in anaerobic environments. In addition, OMVs as biological nano‐heaters for photoacoustic imaging were designed as well. Natural melanin was packaged into OMVs to create biocompatible nanoprobes, which could be applied in non‐invasive monitoring of their spatiotemporal distribution in tumor via multi‐spectral photoacoustic tomography (**Figure** **13**a).^[^ ^112^ ^]^

Along with OMVs, bacterial spores have also received plenty of attention. Various types of Gram‐positive bacteria can form spores under extreme external conditions, which are in spherical or ellipsoidal shape, and naturally wrapped with thick, dense, and hydrophobic coatings. These characteristics endow bacterial spores with resistance against heat, chemical resistance, and radiation.^[^ ^143^ ^]^ Moreover, bacterial spores are dormant and basically thought to be devoid of biological activity, but can germinate to new vegetative cells once the surrounding environment changing to a condition that is suitable for bacterial growth.^[^ ^144^ ^]^ Given these advantages, bacterial spores have been extensively studied and used as carriers in a variety of biomedical applications, including bioimaging and disease therapy.^[^ ^145^, ^146^ ^]^

As a well‐known anti‐cancer bioagent, anaerobic bacteria *C. novyi*‐NT has been found to produce spores that can germinate and proliferate exclusively in hypoxic tumor regions. Similar to live *C. novyi*‐NT, the spores are able to elicit malignant cell lysis and recruit inflammatory cells to tumor site to activate antitumor immune responses. Given the excellent biosafety, these spores have been applied in companion dog studies and human trials.^[^ ^147^, ^148^ ^]^ Moreover, surface decoration has been explored to modify *C. novyi*‐NT spores for visualizing bacterial delivery and biodistribution in cancer therapy. For example, exogenous labeling materials, such as iron oxide nanoparticles, were used to decorate *C. novyi*‐NT spores for MRI, which could then monitor the distribution inside tumor tissue.^[^ ^149^, ^150^ ^]^ In addition, due to the presence of thick protein coatings, bacterial spores are easy to be labeled with a variety of florescent dyes for bioimaging. The spores from probiotic bacterium *Bacillus coagulans* were decorated with deoxycholic acid and chemotherapeutic drugs (doxorubicin and sorafenib) and also labeled with IR780 for both therapy and fluorescence imaging in vivo (Figure 13b).^[^ ^146^ ^]^ With IR780 labeling, the spores were able to emit a strong fluorescence signal and then be tracked in vivo by NIR imaging. Another type of probiotic spores produced by *Clostridium butyricum* has been simultaneously conjugated with gemcitabine‐loaded mesoporous silicon nanoparticles for chemotherapy and Cy5 dye for tumor imaging.^[^ ^151^ ^]^

## 5 Applications of Bacteria‐Based Living Probes

**Figure 14:**
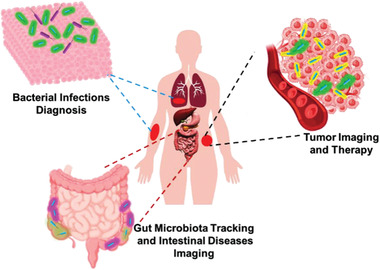
Applications of bacteria‐based living probes in the diagnosis and imaging of bacterial infections, cancers, gut microbiota, and intestinal diseases.

Combining the superior characteristics of bacteria and imageable performance of signal molecules, as well as relying on the continuous development and update of imaging technology and equipment, bacteria‐based living probes exhibit great potential for in vivo applications. In this part, the applications of bacteria‐based living probes in the diagnosis and imaging of bacterial infections, tumors, and intestinal inflammation diseases were highlighted (**Figure** **14**).

### 5.1 Diagnosis of Bacterial Infections

Bacterial infections have been an increasing global problem in modern health care and lead to severe clinical morbidity and mortality.^[^ ^152^ ^]^ In many cases, bacterial infections are typically invisible, which often make patients miss timely treatment.^[^ ^153^ ^]^ Meanwhile, bacterial infections, especially transplantation‐associated infections, are usually accompanied by biofilm formation, which undoubtedly makes treatment more difficult.^[^ ^154^ ^]^ Hence, the development of a sensitive, specific, and non‐invasive method is of great significance for in vivo early‐stage detection of bacterial infections. Currently, the direct labeling of pathogens in the infection sites by imageable probes has been used to detect bacterial infections by performing in vivo bioimaging, which provides an intuitive and feasible solution for infection diagnosis and treatment.^[^ ^155^, ^156^, ^157^ ^]^

**Figure 15:**
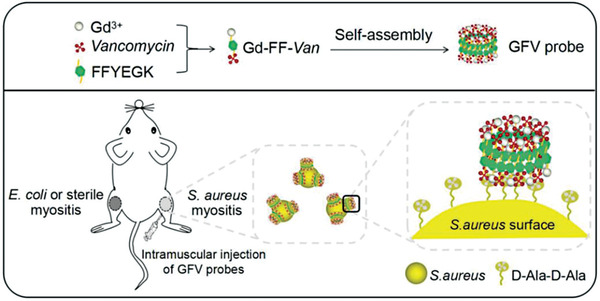
Application of bacteria‐based living probes to detect bacterial infections. T1‐weighted MRI to detect *S. aureus*‐infected myositis. Reproduced with permission.^[^ ^157^ ^]^ Copyright 2021, Wiley‐VCH.

Gram‐negative bacteria, such as *Enterobacterales*, can cause severe, deep‐seated infections, often leading to sepsis or even death. To create a non‐invasive whole‐body analysis tool that can specifically locate pathogens, Jain's group constructed a PET imaging probe on the basis of ^18^F‐FDS, and found that such probes could selectively distinguish *Enterobacterales* infection from other pathologies in mice by PET signal detection.^[^ ^135^ ^]^ Gram‐positive bacterium *S*. *aureus*, one of the main pathogens causing human bacterial infections, generally leads to osteomyelitis, endocarditis and sepsis, resulting in significant clinical and economic burden.^[^ ^155^ ^]^ To avert *S*. *aureus*‐associated infections, it is critical to develop rapid, accurate, and non‐invasive diagnostic methods. For instance, Hernandez et al. designed a bacteria‐based MRI probe by grafting a core superparamagnetic iron oxide nanoparticle (SPION) with *S. aureus*‐specific short oligonucleotide sequences and dendrons functionalized with several gadolinium ion (Gd^3+^) complexes.^[^ ^156^ ^]^ As demonstrated by in vitro imaging of both *S. aureus* and S*taphylococcus epidermidis*, MRI signals could be only detected when *S. aureus* co‐existed with ion. Because in the presence of *S. aureus*, the oligonucleotides on MRI probe could be specifically cleaved by micrococcal nuclease, accompanying by the release of Gd^3+^ complexes from the superparamagnetic SPION core and subsequently the activation of MRI probe.^[^ ^156^ ^]^ Likewise, inspired by the self‐assembly of supramolecular assemblies, Song's group designed and synthesized a T1‐weighted MRI nanoaggregate probe, consisting of vancomycin as a targeting ligand, Gd^3+^ as a T1 signaling unit, and self­assembling peptide FFYEGK for signal enhancement, and tested the specific recognition of probe to *S. aureus* both in vitro and in vivo. Supporting these positive data from *S. aureus*‐induced mouse model of myositis, they proved that such an MRI probe presented the potential for specific and sensitive *S. aureus* infection detection (**Figure** **15**).^[^ ^157^ ^]^ In addition to MRI probes, Cai et al. synthesized a type of core‐shell down‐conversion nanoparticles covalently linked with vancomycin.^[^ ^158^ ^]^ This probe showed excellent emission intensity in the second NIR‐II and a high degree of selectivity to Gram‐positive bacteria. Once this probe was injected into *S. aureus/E. coli* co‐infected mice, a strong NIR‐II signal was clearly detected only in *S. aureus* infected sites during 6 to 24 h post‐injection.^[^ ^158^ ^]^

### 5.2 Tumor Imaging and Therapy

In addition to bacterial infections, the development of imaging probes to detect solid tumors is another important task. During the past decades, unremitting efforts in exploring effective diagnostic and imaging modalities have been paid to eliminate malignant tumors. Interestingly, since W. Busch and W. Coley conducted bacteria‐mediated cancer therapy in the late 19th century, the ability of bacteria to target and colonize tumors has attracted more and more attention. In particular, a wide variety of symbiotic bacteria have been discovered in solid tumors in recent years.^[^ ^159^ ^]^ Therefore, bacteria‐mediated cancer theranostic (BMCT) has become a promising and increasingly popular strategy.^[^ ^159^, ^160^ ^]^

**Figure 16:**
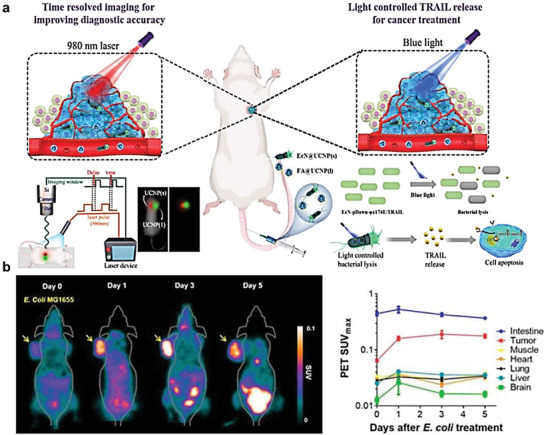
Application of bacteria‐based living probes for tumor imaging and therapy. a) BMCT basing on upconversion optogenetic bacteria‐based living probes. Reproduced with permission.^[^ ^161^ ^]^ Copyright 2019, American Chemical Society. b) ^18^F‐FDS‐assisted PET imaging of *E. coli* MG1655 in tumor‐bearing mice. Reproduced with permission.^[^ ^136^ ^]^ Copyright 2020, Ivyspring International Publisher.

To prepare a living agent for tumor theranostic, Liu et al. constructed a dully modified bacterium capable of producing melanin (Bac^Mel^), with surface decoration of fluorescein isothiocyanate‐labeled immune checkpoint inhibitors of anti‐programmed death‐1 antibodies (αPD‐1) by in situ polymerization of dopamine.^[^ ^160^ ^]^ Relying on the fluorescent signals and specific accumulation of *E. coli* in tumors, as monitored by fluorescence imaging, the modified bacteria could colonize the hypoxia intratumoral microenvironment and be spatiotemporal distributed in solid tumors. Later, they constructed a surface decorated bacteria‐based living probe by covalent attachment of Cy5‐labled aptamers through an amide condensation reaction.^[^ ^52^ ^]^ As demonstrated, thanks to the targeting ability of aptamers, the decorated bacteria facilitated the localization at tumor sites, which could be visualized through in vivo imaging system.^[^ ^52^ ^]^ Besides, photothermal agents such as magnetic nanoparticles and upconversion nanoparticles (UCNP) have been integrated with bacteria for BMCT. Wang et al. constructed a light‐controlled engineered bacterial system basing on UCNP‐mediated time‐resolved imaging (TRI) for the diagnosis of colorectal cancer.^[^ ^161^ ^]^ These bacteria could target the tumor site, realize the co‐localization with tumor tissue, and improve the diagnostic accuracy using TRI. In addition, blue light irradiation could induce the cleavage of engineered bacteria and subsequently enable the release of tumor apoptosis‐related induction ligands, which could trigger tumor cell death (**Figure** **16**a).^[^ ^161^ ^]^ Radiotracer‐labeled bacteria‐based living probes represent another type of important living bioagents that can be applied for tumor imaging. Blasberg et al. prepared two radiotracers [^18^F]−2′‐Fluoro‐2′deoxy‐1h‐D‐arabionofuranosyl‐5‐ethyl‐uracil (^18^F‐FEAU) and [^124^I]−2′‐fluoro‐1‐h‐D‐arabino‐furanosyl‐5‐iodo‐uracil (^124^I‐FIAU), and investigated the feasibility of non‐invasive PET imaging for EcN‐colonized tumors.^[^ ^162^ ^]^ They found that EcN could selectively phosphorylate pyrimidine nucleoside analogues and then trap them efficiently. Compared to ^18^F‐FDG, ^18^F‐FEAU provided improved PET imaging (higher signal‐to‐background ratio) in EcN‐colonized tumors.^[^ ^162^ ^]^ Moreover, Min et al. synthesized a radioactive compound of ^18^F‐FDS, which was able to target *E. coli* in tumor site, and the distribution of these bacteria inside the tumor tissue could be observed by PET imaging shortly after injection.^[^ ^136^ ^]^ This method overcame the limitation of tissue penetration depth in optical imaging and paved an avenue for the visualization and monitoring of bacteria‐based tumor treatment (Figure 16b).^[^ ^136^ ^]^

### 5.3 Bioimaging of the Gut Microbiota and Intestine‐Associated Diseases

As the gut microbiota plays critical roles in maintaining mammalian homeostasis and host health, real‐time, and easy‐operate tracking of the gut microbiota in vivo is of great significance for examining their distribution and colonization, and understanding their behaviors and biological functions.^[^ ^163^ ^]^ Thus, the imaging technology of bacteria‐based living probes endows us with more opportunities to understand the gut microbiota in a directly visual way.

**Figure 17:**
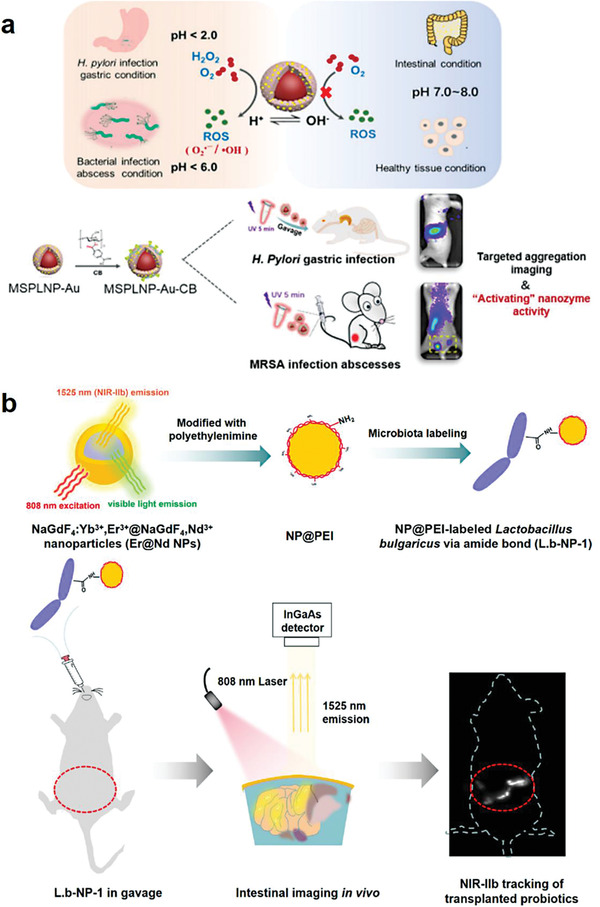
Application of bacteria‐based living probes in intestinal disease diagnosis and therapy. a) Luminescence imaging of *H. pylori* in the intestine. Reproduced with permission.^[^ ^165^ ^]^ Copyright 2021, American Chemical Society. b) NIR imaging of probiotics in the intestine. Reproduced with permission.^[^ ^166^ ^]^ Copyright 2023, American Chemical Society.

Bacteria‐based living probes with surface‐modified fluorescent molecules or intracellularly expressed fluorescent proteins are extremely convenient to track the colonization and localization of bacteria in the gut. Han et al. reported a SYTO‐9‐labeled bacteria‐based probe that could be electrostatically encased in a colloidal shell composed of amino‐modified poly‐β‐cyclodextrin and tannic acid, which enhanced oral availability and prolonged the intestinal adhesion duration, showing by in vivo imaging.^[^ ^164^ ^]^ Liu et al. encapsulated GFP‐expressing bacteria with a lipid membrane coating through interfacial supramolecular self‐assembly to build a bacteria‐based probe, with abilities to fight against various harsh environments, such as gastric acid and antibiotics, as well as to visualize the colonization of bacteria in the intestinal tract in vivo via fluorescent imaging.^[^ ^40^ ^]^ *H. pylori* is a bacterium with high adaptability in extreme environments and can cause different diseases, such as peptic ulcers, gastric ulcers, and gastric cancer.^[^ ^54^ ^]^ Yan et al. prepared a pH‐responsive persistent luminescence nanozyme (MSPLNP‐Au‐CB), which could especially target both *H. pylori* and methicillin‐resistant *S. aureus* for in vivo bioluminescence imaging and inactivation under acidic conditions (**Figure** **17**a).^[^ ^165^ ^]^

Despite the long duration and strong penetration ability, the broad application of bacteria‐based fluorescent probes has been inevitably impeded. NIR fluorescent proteins or dyes, especially the ones in NIR‐II region, have been considered as a superior alternative for intestinal imaging and corresponding diagnosis. For example, Cai et al. constructed a bacteria‐based probe via covalent labeling on the surface of *Lactobacillus bulgaricus* for in vivo NIR‐IIb imaging.^[^ ^166^ ^]^ According to the data from NIR‐Iib imaging, higher spatiotemporal resolution for the tracking and visualization of the localization and distribution of transplanted bacteria in the intestine was achieved (Figure 17b).^[^ ^166^ ^]^ In addition, Tan's group labeled the gut microbiota with NIR‐II dye by click chemistry, revealing high spatial resolution and deep tissue penetration in mice via NIR fluorescent imaging.^[^ ^167^ ^]^

Another two non‐invasive and real‐time imaging technologies with enhanced penetration depth, MRI, and PET imaging, have been commonly used to monitor the dynamic behavior of the gut microbiota in vivo. Gao et al. used ^19^F metabolism to label the gut microbiota and realized real‐time ^19^F MRI in vivo imaging and the extended visualization of gut microbes in different intestinal segments.^[^ ^168^ ^]^ Liu et al. labeled *B. fragilis* with ^64^Cu and fluorescent dye through metabolic oligosaccharide engineering and biorthogonal click chemistry, and successfully detected the in vivo behavior of *B. fragilis* after transplantation by PET imaging.^[^ ^133^ ^]^ These strategies provide a valuable basis for in situ imaging and real‐time monitoring of the gut microbiota, representing a favorable reference for the development of novel bacteria‐based living probes for future clinical applications.

## 6 Conclusion and Outlook

Bioimaging provides a powerful means to understand the structure of biological tissues and elucidate various physiological functions of organisms. Thanks to the rapid development of imaging technologies, bioimaging has made rapid progress and received dramatic attention, especially in the field of biomedicine for initial diagnostic imaging of diseases. To achieve more sensitive and accurate bioimaging and diagnosis in a real‐time and non‐invasive manner, various types of probes have been developed. In this review, we have briefly summarized the design principles of bacteria‐based living probes, mainly including biological engineering, chemical modification, intracellular loading, and optical manipulation, and systemically introduced the latest progress for fluorescence imaging, NIR imaging, ultrasonic imaging, photoacoustic imaging, MRI, and PET imaging. Additionally, the applications of these living probes in bioimaging, diagnosis, and even treatment of various diseases, such as bacterial infections, tumors, and intestinal diseases have been highlighted. Although significant progress of bacteria‐based living probes has been made in preclinical studies, due to the limitations of both bacteria (such as dose‐dependent side effects, uncontrolled colonization, and unfavorable distribution) and imageable agents, numerous challenges need to be addressed before considering for further translation. To this end, the development of superior living probes with satisfied properties of specific accumulation, high spatial resolution, and large tissue penetration depth are highly desirable for bioimaging and diagnosis of various diseases. In summary, this review article offers an updated overview of bacteria‐based living probes, spotlighting their promising potential as a unique yet universal platform for designing next‐generation imageable agents. We anticipate the use of bacteria‐based living probes for clinical applications in intelligent bioimaging, diagnosis, and even therapy in the near future.

## Conflict of Interest

The authors declare no conflict of interest.
